# DISCORD BETWEEN SELF-REPORT AND PERFORMANCE-BASED ADL MEASURES AMONG PREFRAIL OR FRAIL OLDER ADULTS

**DOI:** 10.1093/geroni/igae098.2604

**Published:** 2024-12-31

**Authors:** Chiung-ju (C J) Liu, Wen-Pin Chang, Inga Wang

**Affiliations:** University of Florida, Gainesville, Florida, United States; The University of Texas Rio Grande Valley, Edinburg, Texas, United States; University of Wisconsin Milwaukee, Milwaukee, Wisconsin, United States

## Abstract

Prefrail and frail older adults face an increased risk of late-life disability. Detecting the decline in activities of daily living is essential to mitigate or reverse the progression of disability in this population. However, self-report and performance-based measures of activities of daily living do not always coincide because the magnitude of decline could be too subtle to detect or be aware of. This study examined the correlation between the two evaluation approaches and compared the characteristics of participants who overestimated with those who underestimated their performance. The baseline data of 71 prefrail or frail, community-living older adults (Mean age = 74 years) from previous clinical trials were analyzed. There was no statistically significant correlation between the self-reported measure in activities of daily living (assessed by the Performance Score of the Canadian Occupational Performance Measure) and the performance-based measure (assessed by the Motor Skills Score of the Assessment of Motor and Process Skills), rs =.01, p =.94. Approximately half of the participants showed a discrepancy between the two measures. Those who overestimated (n = 12) tended to be older and had poorer physical functioning than those who underestimated (n = 20), all ps <.05. Additionally, those who overestimated were more satisfied with their performance of activities of daily living than those who underestimated it, Z = 35.55, p <.05. Overestimation and underestimation may reflect different coping mechanisms adopted by prefrail and frail older adults in response to functional decline. Research to further investigate these mechanisms is encouraged.

